# Tackling health inequalities together: inclusion health and co-production

**DOI:** 10.3399/bjgp24X739365

**Published:** 2024-09-27

**Authors:** Helen McGeown, Lucy Potter, Danny Sherwood, Bridging Gaps group, Jeremy Horwood, Cait Taylor, Michelle Farr

**Affiliations:** Centre for Academic Primary Care, Population Health Sciences, Bristol Medical School, University of Bristol, Bristol.; Centre for Academic Primary Care, Population Health Sciences, Bristol Medical School, University of Bristol, Bristol.; Lead, Co-create, Sheffield.; Lived-experience experts.; Professor of Social Science and Health, Centre for Academic Primary Care, Population Health Sciences, Bristol Medical School, University of Bristol, Bristol; National Institute for Health and Care Research Applied Research Collaboration West, University Hospitals Bristol and Weston NHS Foundation Trust, Bristol.; Central Liverpool Primary Care Network, Liverpool.; Senior Research Fellow in Health Research, Centre for Academic Primary Care, Population Health Sciences, Bristol Medical School, University of Bristol, Bristol; National Institute for Health and Care Research Applied Research Collaboration West, University Hospitals Bristol and Weston NHS Foundation Trust, Bristol.

## Introduction

Trauma-informed approaches acknowledge the impact of trauma on the health of patients and staff, and the ways in which health care is accessed and experienced.^1^ There is growing interest in these approaches in both a UK and international primary care context.^2^ Intrinsic to a trauma-informed approach (although rarely achieved in practice)^2^ is the co-production of health services with those with relevant lived experience.

Existing models of patient involvement in primary care tend to rely significantly on feedback forms or surveys, which do not facilitate an ongoing dialogue between patients and healthcare providers. Patient participation groups (PPGs) can provide a more meaningful space in which services can be truly co-produced, in particular where careful attention is given to power dynamics in the room.^3^ There is, however, an increasing understanding of the need for flexibility in our approaches to patient involvement, which should incorporate multiple models, recognising individuals’ diverse experiences, preferences, and capacity for participation.^3^ The Care Quality Commission (CQC) suggests that we may have *‘… more effective conversations through engaging with different community groups at different times and in different places’*.^4^ The need for provision of protected spaces with careful attention to sociocultural backgrounds and power dynamics is echoed within the principles of trauma-informed care.^1^

The current authors have worked proactively with specific community groups who are often poorly served by our health services. We have sought to create protected spaces and mitigate existing power dynamics to support the free communication of ideas, working together with partnership organisations to improve local primary care provision.

## Case study 1: Bridging Gaps

One25 is a third-sector organisation in Bristol that supports women with severe and multiple disadvantage (SMD — including combinations of homelessness, substance misuse, street-based sex work, violence and abuse, and poor mental health). These women have all encountered significant chronic and complex trauma, often since childhood. Delivering a clinic at One25’s drop-in centre, Dr Lucy Potter was struck by many unjust examples of the inverse care law where those most in need of health care had the poorest access to it.^7^ Despite efforts from support staff, mainstream general practices were inaccessible to women who attended the drop-in centre. Together with the women and staff from One25 and academic colleagues working in health care, she established ‘Bridging Gaps’.^8^^,^^9^ Group membership, activities, and learning are outlined in Box 1. Developing a partnership between several organisations was key to providing diverse skill-sets needed for co-production, and our full methodology is outlined in our prior publications for those interested in further details.^8^^,^^9^

**Box 1.** Case study 1

**Project name**	**Bridging Gaps**
Location	Bristol
Partnership organisations	Women with lived experience of severe and multiple disadvantage from One25 charity, One25 staff, researchers from National Institute for Health and Care Research Applied Research Collaboration West and Centre for Academic Primary Care, University of Bristol, GPs, nurses, practice managers, receptionists, and care coordinators, Bristol, North Somerset, South Gloucestershire Integrated Care Board (ICB).
Group activities	Fortnightly funded meetings for women with lived experience of severe and multiple disadvantage in a community centre to share experience, build skills and confidence, and co-produce strategy for change, co-facilitated by a specialist inclusion health GP (Dr Lucy Potter), a charity support worker, and a healthcare researcher. Supporting women with lived experience to participate in collaborative service change meetings with general practice staff to better support people with trauma and multiple disadvantage. Working with other organisations who also want to improve access to health care, for example, the local ICB and Pathway (a national homeless and inclusion health charity) to share experience and insights from the changes developed and implemented so far.
Outputs	Each practice had a bespoke plan for service development by the end of Bridging Gaps’ involvement. Changes enacted included: development of an enhanced access list for patients with severe and multiple disadvantage; care coordinators given a dedicated role in facilitating access for those on the enhanced access list; dedicated inclusion health clinic (with proactive identification of patients not currently reached); trauma-informed training for practice staff; and a personal snapshot document (allowing patients to provide background details for their records — avoiding re-telling traumatic experiences). Other outputs included training on co-production, website development (https://bridginggaps.blogs.bristol.ac.uk/), the ‘top tips for trauma-informed primary care’ document (available on request from corresponding author), and training materials for GP trainees.
Recommendations/key learning	Co-production work is resource intensive and needs sufficient time and expertise to be carried out safely and properly. Partnership working with organisations is key. Our group included facilitators with expertise in co-production, in supporting trauma survivors, and in primary care. When working with people who have experienced trauma, there is a need for support during and between group meetings. Check-ins and check-outs at meetings are paramount to understand how participants are feeling, because some meetings can be triggering. Breaks within and care after meetings are key as heavy subjects can come up (trauma can affect everyone). Providing a thank you to participants shows value and worth. Payment guidance is available.^5^ Trust is vital. Sufficient time is key to enable trust to be established within the group. We took a bespoke approach as different things worked at different GP practices. Working with care coordinators who can have a more personal relationship with patients was particularly helpful. When things did not work, we found this to be a way of learning, so it should not be negative or seen as failure but valuable information. Groups need mutually agreed ground rules and facilitators need to fairly apply these to all within the group. We are equal, so we share decision making, with no ‘them and us’ vibe. We listen to each other, and all views and inputs are valued. Sharing food and drinks around a table, with everyone having a common goal, can break barriers and remove any hierarchy. Those with lived experience are there to share their expertise rather than necessarily their difficult experiences. It is important that no one feels under any pressure to share difficult or traumatic experiences.

## Case study 2: Tackling Racial Inequalities in Health

Central Liverpool Primary Care Network (CLPCN) is a relatively large PCN of 129 000 patients. Group membership, activities, and learning are outlined in Box 2. It is the most ethnically diverse PCN in Liverpool with 29.2% of the population coming from global majority communities. There are well-established data showing increased disease prevalence and poor health outcomes in these groups. All PCNs have a remit to tackle health inequality and CLPCN has established a specific priority to tackle racial inequality. The CLPCN Tackling Racial Inequality Working Group (TRIWG), led by GPs, Dr Fiza Salam and Dr Cait Taylor, was set up in July 2020 as a response to the Black Lives Matter movement and the mounting evidence for the disproportionate impact of COVID-19 on global majority communities due to social and economic inequalities, racism, discrimination, occupational risk, and inequalities in the surveillance of long-term conditions. Lack of trust in NHS services and healthcare treatment was also identified as a key issue.

**Box 2.** Case study 2

**Project name**	**Tackling Racial Inequalities in Health**
Location	Liverpool
Partnership organisations	Central Liverpool Primary Care Network engagement leads, clinical director, GPs, practice managers, practice administrative staff, social prescribers, voluntary sector representatives, community activists, and staff from Co-create — a consultancy organisation that supports the use of co-production and related approaches to develop fairer and more effective public services.
Group activities	Monthly 3-hour sessions, including asset mapping. Creation of multiple engagement pilot sub-groups, which received 1:1 support from Co-create to engage with members from diverse communities. Outreach activities included: coffee mornings at a community centre for Irish Travellers; face-to-face groups with refugees and asylum-seeking people at local community centres; drop-in sessions at a community hub for young people; conversations with Chinese community groups; social media campaigns targeting local young people; and face-to-face and telephone interviews with local older Muslim women.
Outputs	Map of existing groups and other resources relevant to tackling racial inequalities, and development of relationships with some of these organisations. Resources in multiple languages to help people navigate the local NHS services. Language ID cards for reception staff to use, which have written language, country flags, and the pin code for language line translation services. Primary care network and practice staff training including use of the Safe Surgeries Toolkit.^6^ Work to simplify and unify the GP registration pathway. Patient involvement in improving ethnicity recording. Increased community representation in the primary care network. A strategy to improve diversity of primary care network and practice staff. Continuous engagement, partnership, and work towards a city-wide Racism Truth Commission.
Recommendations/key learning	Co-production work takes time. This should be planned for from the outset, regarding both individual session length and overall duration of the project. Include trust-building exercises at the start of co-production processes. Many individuals had very little trust in medical professionals at the beginning of the process. Going to where people already are and being flexible in modes, timing, and location supports a wider range of patients to engage. Many individuals were happy to engage but the practicalities of arranging and attending meetings acted as a barrier. Training on active listening would be helpful for those doing the engagement — especially for GPs who are trained in trying to give clear-cut answers. Be aware of the risk of overwhelm when dealing with topics related to health inequalities, and of the emotional load involved in group participation/facilitation. Provide reflection time for those involved in engagement.

CLPCN commissioned Co-create to assist them in involving their patients in service development and identifying and addressing barriers to health equality.

## Reflections

Our experiences affirm calls from the CQC for innovation, flexibility, and provision of ‘safe spaces’ based on protected characteristics. The Bridging Gaps group was exclusively female, and this helped many to share difficult experiences of health care relating to gender. One group member said: *‘Women together … we have a bit of a giggle … it feels like a safe space’* (Lived-experience member 1).^8^ The TRIWG considered how to engage with individuals from a range of backgrounds, often opting to ‘join in’ with existing services to meet people from specific racial or ethnic groups where they already gather. Both groups provided flexibility in modes of engagement, including in person and online, and participants could join in and out of group activities as their life circumstances changed.

As evidenced by Box 1 and Box 2, there was significant overlap in the key learning from both groups’ experiences. It is our hope that this learning can benefit others wishing to embark on similar work, and indeed perhaps inform future guidance on patient participation. An over-riding finding from both projects was that trust is vital for co-production to work. This takes time to grow and must be actively cultivated, which requires longer timeframes than other service development projects. Our experiences highlighted risks of triggering and emotional overwhelm through group participation for both staff and patients. To mitigate this risk, it should be clearly communicated that there is no pressure or expectation that details of personal or traumatic experiences be shared within a co-production group. People are free to share what they wish. In both projects, working with existing community groups outside of general practice who have relevant expertise helped considerably in managing power dynamics within meetings and providing support between them. Establishing mutually agreed roles and boundaries for the group should be an early group activity, and decisions should be made from project commencement on how agreed boundaries are to be upheld. This includes careful consideration and clear communication around safeguarding and maintaining confidentiality. Safety can be further maintained by seeking to include professionals from the same gender, ethnic, or cultural backgrounds as those with lived experience within co-production groups.

The need for significant support, time, resource, and expertise to enable safe participation for those who are least well served by existing models of care may in part explain why so many voices are currently excluded from development of primary care services. This will likely perpetuate health inequalities. Our experiences highlight that co-production is possible, but collaboration is key. To move beyond the PPG, practices need to look to their wider communities and ‘join in’ with what is already going on. As demonstrated in Liverpool, outreach by local PCNs to community groups can greatly widen participation in service development. Partnering with charity One25 in Bristol gave Bridging Gaps access to those with the experience and availability to provide regular support to members of our group. As we seek to move towards the delivery of trauma-informed care, there is a need for dedicated resource and funding so that the patient voice is meaningfully and safely embedded at the heart of future system change.
